# Supplementary material for: Tuning the Fermi velocity in Dirac materials with an electric field
Source: Sci Rep. 2017 Aug 14;7:8058. doi: 10.1038/s41598-017-08188-3 (PMC5556122; doi:10.1038/s41598-017-08188-3)
Supplement: Supplementary file 1 — Supplemental Material: [file 41598_2017_8188_MOESM1_ESM.pdf]

Supplemental Material: Tuning the Fermi velocity in Dirac materials with an electric field

A. Díaz-Fernández,[1,2] Leonor Chico,[3,4] J. W. González,[4,5] and F. Domínguez-Adame[1,2]

[1] *GISC, Departamento de Física de Materiales, Universidad Complutense, E-28040 Madrid, Spain*

[2] *Department of Physics, University of Warwick, Coventry, CV4 7AL, United Kingdom*

[3] *Instituto de Ciencia de Materiales de Madrid, Consejo Superior de Investigaciones Científicas, C/ Sor Juana Inés de la Cruz 3, E–28049 Madrid, Spain*

[4] *Donostia International Physics Center, Paseo Manuel de Lardizabal 4, E–20018 Donostia–San Sebastián, Spain*

[5] *Centro de Física de Materiales (CSIC-UPV/EHU)-Material Physics Center (MPC), Paseo Manuel de Lardizabal 5, E–20018 Donostia–San Sebastián, Spain*

EXACT SOLUTION TO A SYMMETRIC BAND-INVERTED JUNCTION IN A UNIFORM ELECTRIC FIELD

Here we solve the problem without making use of Feynman-Gell-Mann *ansatz* and for arbitrary values of the electric field. We begin by considering the envelope-function for a symmetric-gap junction in a uniform electric field along the z-direction, i.e., the growth direction. It satisfies the following Dirac-like equation

$$\left[-i\hbar v_z\alpha_z\frac{\mathrm{d}}{\mathrm{d}z}+\hbar v_\perp\boldsymbol{\alpha}_\perp\cdot\mathbf{k}_\perp+\Delta\mathrm{sgn}(z)\beta-(E+eFz)\right]\boldsymbol{\chi}(z)=0\ , \tag{S1}$$

with the same notation as in the main text. Using the reduced variables introduced therein, namely $d=\hbar v_z/\Delta$, $\xi=z/d$, $\boldsymbol{\kappa}=(v_\perp d/v_z)\mathbf{k}_\perp$, $\epsilon=E/\Delta$ and $f=eFd/\Delta$, and performing a change of basis with $\mathcal{P}=2^{-1/2}\left[\beta+\sigma_x\otimes\mathbb{1}_2\right]$, so $\boldsymbol{\Phi}=\mathcal{P}\boldsymbol{\chi}$, we arrive at

$$\begin{pmatrix}\partial_\xi^2-if+(\epsilon+f\xi)^2-\kappa^2-1 & 0\\ 0 & \partial_\xi^2+if+(\epsilon+f\xi)^2-\kappa^2-1\end{pmatrix}\boldsymbol{\Phi}^u=0\ , \tag{S2}$$

where $\boldsymbol{\Phi}=\left(\boldsymbol{\Phi}^u,\boldsymbol{\Phi}^d\right)^T$. We solve the problem for $\boldsymbol{\Phi}^u$ and then $\boldsymbol{\Phi}^d$ can be obtained from

$$\begin{pmatrix}-i\partial_\xi-(\epsilon+f\xi) & \kappa e^{-i\theta_\kappa}\\ \kappa e^{i\theta_\kappa} & i\partial_\xi-(\epsilon+f\xi)\end{pmatrix}\boldsymbol{\Phi}^u=-s(\xi)\boldsymbol{\Phi}^d\ , \tag{S3}$$

where $\kappa=|\boldsymbol{\kappa}|$ and $\theta_\kappa=\tan^{-1}(k_y/k_x)$.

Let $x=(\epsilon+f\xi)/\sqrt{f}$ and $\mu^2=(\kappa^2+1)/f$. Then it can be shown straightforwardly that

$$\boldsymbol{\Phi}(x)=\begin{cases}\beta\mathcal{F}(x)\,\boldsymbol{C}_+\ , & \xi>0\ ,\\ \mathcal{F}(x)\,\boldsymbol{C}_-\ , & \xi<0\ .\end{cases} \tag{S4}$$

Here $\boldsymbol{C}_\pm$ are two constant vectors and

$$\mathcal{F}(x)=\begin{pmatrix}F^*(x) & G(x) & 0 & 0\\ 0 & 0 & F(x) & G^*(x)\\ \sqrt{\kappa^2+1}\,G^*(x) & \sqrt{\kappa^2+1}\,F(x) & \kappa e^{-i\theta_\kappa}F(x) & \kappa e^{-i\theta_\kappa}G^*(x)\\ \kappa e^{i\theta_\kappa}F^*(x) & \kappa e^{i\theta_\kappa}G(x) & \sqrt{\kappa^2+1}\,G(x) & \sqrt{\kappa^2+1}\,F^*(x)\end{pmatrix}\ , \tag{S5}$$

being $F(x)$ and $G(x)$ given by [**?**]

$$F(x)=M\left(-i\frac{\mu^2}{4},\frac{1}{2},ix^2\right)e^{-ix^2/2}\ ,\qquad G(x)=i\mu xM\left(1-i\frac{\mu^2}{4},\frac{3}{2},ix^2\right)e^{-ix^2/2}\ , \tag{S6}$$

where $M(a,b,z)$ are Kummer's functions [**?**].

The problem can now be solved by considering continuity at $z = 0$ and applying boundary conditions at $\pm\infty$. Although the former is easily applied, the latter are not so straightforward. In order to account for those conditions, we consider our system placed within a very large box in the z-direction, such that the outward component of the current density can be set to zero. Considering a box of size $2L$ and $L \gg d$, the condition for a vanishing current amounts to [?]

$$i\beta\alpha_z \boldsymbol{\chi}(x_-) = \boldsymbol{\chi}(x_-) \ , \qquad -i\beta\alpha_z \boldsymbol{\chi}(x_+) = \boldsymbol{\chi}(x_+) \ , \tag{S7}$$

where we define $x_\pm = (\epsilon \pm fd/L)/\sqrt{f}$. By considering this prescription and continuity at $z = 0$ we obtain the desired implicit relationship between ϵ and κ for any value of the reduced field f

$$\det \left[\mathcal{N}_- + \mathcal{N}_+ \mathcal{F}_0^{-1} \beta \mathcal{F}_0 \right] = 0 \ , \tag{S8}$$

with $\mathcal{F}_0 = \mathcal{F}(x_0)$ and $x_0 = \epsilon/\sqrt{f}$. The matrices $\mathcal{N}_\pm$ are given by

$$\mathcal{N}_+ = \begin{pmatrix} \mathcal{M}_+ \\ \mathbb{O}_{2\times 4} \end{pmatrix} \ , \qquad \mathcal{N}_- = \begin{pmatrix} \mathbb{O}_{2\times 4} \\ \mathcal{M}_- \end{pmatrix} \ , \tag{S9}$$

where

$$\mathcal{M}_\pm = \begin{pmatrix} F_\pm^* + i\sqrt{\kappa^2+1}G_\pm^* & G_\pm + i\sqrt{\kappa^2+1}F_\pm & i\kappa e^{-i\theta_\kappa} F_\pm & i\kappa e^{-i\theta_\kappa} G_\pm^* \\ -i\kappa e^{i\theta_\kappa} F_\pm^* & -i\kappa e^{i\theta_\kappa} G_\pm & F_\pm - i\sqrt{\kappa^2+1}G_\pm & G_\pm^* - i\sqrt{\kappa^2+1}F_\pm^* \end{pmatrix} \ , \tag{S10}$$

with $F_\pm = F(x_\pm)$ and $G_\pm = G(x_\pm)$.

Equation (??) cannot be reduced any further, and an analytic expression as the one shown in the main text [see Eq. (8a)] is unobtainable. The numerical solution of Eq. (??) at $L \gg d$ allows us to verify that the in-plane dispersion is still a Dirac cone that becomes wider upon increasing the electric field. Additionally, we can numerically confirm that the results given by this full approach match the low-field limit presented in the main text, as Fig. ?? shows. Remarkably, only small deviations of 0.5% to 5.6% are observed in the range $0.05 \leq f \leq 0.3$. This agreement is quite noteworthy and supports the assumptions made in the main text.

RESONANCES AT LOW ELECTRIC FIELD

Approximate solutions to Eq. (7) of the main text can be obtained in closed form in the low-electric-field limit. The argument of the Airy functions is large if $F \ll F_\mathrm{C}$ and we make

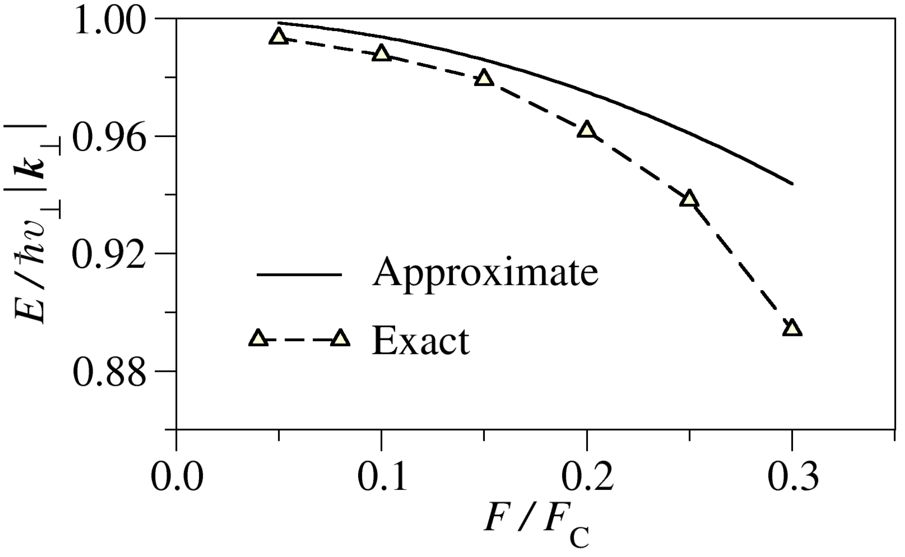

FIG. S1. Reduced Fermi velocity as a function of the reduced electric field obtained from (**??**) (triangles) compared to the approximate result (8a) of the main text (solid line).

use of their asymptotic expansion [**?**]

$$\mathrm{Ai}(z) \simeq \frac{1}{2\sqrt{\pi}}\,\frac{e^{-\phi}}{z^{1/4}}\,L(-\phi)\ , \quad \mathrm{Bi}(z) \simeq \frac{1}{\sqrt{\pi}}\,\frac{e^{\phi}}{z^{1/4}}L(\phi)\ , \tag{S11}$$

with $\phi = (2/3)z^{3/2}$ and

$$L(\phi) = 1 + \sum_{\ell=1}^{\infty}\frac{u_\ell}{\phi^\ell}\ , \qquad u_\ell = \frac{\Gamma(3\ell+1/2)}{54^\ell\,\ell!\,\Gamma(\ell+1/2)}\ , \tag{S12}$$

$\Gamma(z)$ being the Γ function. Since $e^{-4\phi} \ll 1$ one obtains from Eq. (7)

$$\lambda^2 \simeq L^2(\phi)L^2(-\phi)\left[1 + ie^{-2\phi}\,\frac{L(-\phi)}{L(\phi)}\right] \simeq L^2(\phi)L^2(-\phi)\left[1 + ie^{-2\phi}\right]\ . \tag{S13}$$

Keeping terms up to ϕ^{-2} and recalling Eq. (3) one gets

$$\varepsilon_r \simeq \pm\left(1 - \frac{5}{8}\,f^2\right)|\boldsymbol{\kappa}|\ , \qquad \gamma \simeq \frac{1}{|\varepsilon_r|}\,\exp\left(-\frac{2}{3|\varepsilon_r|f}\right)\ , \tag{S14}$$

which give us Eqs. (8a) and (8b).

ARMCHAIR GRAPHENE NANORIBBON WITH AN APPLIED TRANSVERSE ELECTRIC FIELD

Graphene allows for a low-energy description in terms of a massless Dirac equation around the high-symmetry points of the Brillouin zone, $\mathbf{K}$ and $\mathbf{K}'$. Let $\Phi = [\Phi_K, \Phi_{K'}]^T$ with

$\Phi_{K(K')} = \left[\phi_{AK(K')}, \phi_{BK(K')}\right]^T$, where A and B denote the two sublattices. In this basis, the massless Dirac-like Hamiltonian reads

$$H_D = v_F \left(p_x\sigma_x\tau_z + p_y\sigma_y\right) \ , \tag{S15}$$

where $\tau_z = \pm 1$ acts on the valley degree of freedom $\mathbf{K}/\mathbf{K}'$. Let us consider a symmetric armchair nanoribbon of width W along the x direction. Then, a uniform electric field across the ribbon, $\mathbf{F} = F\hat{\mathbf{x}}$ can be modelled by adding an electrostatic potential of the form,

$$V(x) = -eFx + V_0 \ , \qquad V_0 = eF\frac{\tilde{W}}{2} \ , \tag{S16}$$

where $\tilde{W} = W + a$, being $a = \sqrt{3}a_{CC}$ with $a_{CC} = 1.42$ Å the carbon-carbon distance. Due to the translational invariance in the y direction we can ask for solutions of the form $\Phi_K(\mathbf{r}) = \exp(ik_y y)\Phi(x)$. This problem can be exactly solved using the following boundary conditions [**? ?**]

$$\Phi_K(x=0) = -\Phi_{K'}(x=0) \ , \qquad \Phi_K(\tilde{W}) = -\Gamma\Phi_{K'}(\tilde{W}) \ , \tag{S17}$$

with $\Gamma = \exp(-2\pi i 4\tilde{W}/3a)$. It is not difficult to show that metalic nanoribbons take place whenever $\Gamma = 1$, that is, when $4\tilde{W}/3a = n$ with $n \in \mathbb{N}$.

In order to solve the problem, it is more convenient to turn to non-dimensional variables. Let

$$\xi = \frac{x}{\tilde{W}} - \frac{1}{2} \ , \qquad k = \tilde{W}\hbar k_y, \qquad f = \frac{F}{F_W} \ , \epsilon = \frac{E\tilde{W}}{\hbar v_F} \ , \tag{S18}$$

where $F_W = \hbar v_F / e\tilde{W}^2$. Then Dirac equation is written as

$$\left[-i\sigma_x\tau_z\partial_\xi + k\sigma_y\right]\Phi(\xi) = \left(f\xi + \epsilon\right)\Phi(\xi) \ . \tag{S19}$$

where $\partial_\xi \equiv \partial/\partial\xi$.

Solving the Dirac equation, together with the boundary conditions for a metallic nanoribbon, we get to the following implicit equation for the energy

$$1 - \Re\left[(F_+^2 - G_+^2)(F_-^{*2} - G_-^{*2})\right] + 4\Im\left[F_+G_+^*\right]\Im\left[F_-G_-^*\right] = 0 \ , \tag{S20}$$

where $F_\pm = F(z_\pm)$ and $G_\pm = G(z_\pm)$, being

$$F(z) = M\left(-i\mu^2, \frac{1}{2}, iz^2\right)e^{-iz^2/2} \ , \qquad G(z) = 2i\mu z M\left(1 - i\mu^2, \frac{3}{2}, iz^2\right)e^{-iz^2/2} \ , \tag{S21}$$

and

$$\mu = \frac{k}{2\sqrt{f}} \ , \qquad z_\pm = \frac{1}{\sqrt{f}}\left(\epsilon \pm \frac{f}{2}\right) \ . \tag{S22}$$

[S] F. Sauter, Z. Physik **69**, 742 (1931).

[S] M. Abramowitz and I. Stegun, *Handbook of Mathematical Functions* (Dover, New York, 1972).

[S] P. Alberto, S. Das, and E. C. Vagenas, Phys. Lett. A **375**, 1436 (2011).

[S] L. Brey and H. A. Fertig, Phys. Rev. B **73**, 235411 (2006).

[S] J. Wurm, M. Wimmer, I. Adagideli, K. Richter, and H. U. Baranger, New J. Phys. **11**, 095022 (2009).
